# Winter Soil CO_2_ Flux from Different Mid-Latitude Sites from Middle Taihang Mountain in North China

**DOI:** 10.1371/journal.pone.0091589

**Published:** 2014-03-10

**Authors:** Huitao Shen, Jiansheng Cao, Wanjun Zhang, Xinhua Zeng, Huaru Wang

**Affiliations:** 1 Key Laboratory for Agricultural Water Resources, Hebei Key Laboratory for Agricultural Water-Saving, Center for Agricultural Resources Research, Institute of Genetics and Developmental Biology, Chinese Academy of Sciences, Shijiazhuang, Hebei, China; 2 Yellow River Water Resources Protection Institute, Zhengzhou, He’nan, China; DOE Pacific Northwest National Laboratory, United States of America

## Abstract

Winter soil respiration is a very important component of the annual soil carbon flux in some ecosystems. We hypothesized that, with all other factors being equal, shorter winter SR result in reduced contribution to annual soil C flux. In this study, the contribution of winter soil respiration to annual soil respiration was measured for three sites (grassland: dominated by *Artemisia sacrorum*, *Bothriochloa ischaemum* and *Themeda japonica*; shrubland: dominated by *Vitex negundo var. heterophylla*; plantation: dominated by *Populus tomatosa*) in a mountainous area of north China. Diurnal and intra-annual soil CO_2_ flux patterns were consistent among different sites, with the maximum soil respiration rates at 12∶00 or 14∶00, and in July or August. The lowest respiration rates were seen in February. Mean soil respiration rates ranged from 0.26 to 0.45 µmol m^−2^ s^−1^ in the winter (December to February), and between 2.38 to 3.16 µmol m^−2^ s^−1^ during the growing season (May-September). The winter soil carbon flux was 24.6 to 42.8 g C m^−2^, which contributed 4.8 to 7.1% of the annual soil carbon flux. Based on exponential functions, soil temperature explained 73.8 to 91.8% of the within year variability in soil respiration rates. The Q_10_ values of SR against ST at 10 cm ranged from 3.60 to 4.90 among different sites. In addition, the equation between soil respiration and soil temperature for the growing season was used to calculate the “modeled” annual soil carbon flux based on the actual measured soil temperature. The “measured” annual value was significantly higher than the “modeled” annual value. Our results suggest that winter soil respiration plays a significant role in annual soil carbon balance, and should not be neglected when soil ecosystems are assessed as either sinks or sources of atmospheric CO_2_.

## Introduction

Soil respiration (SR) provides the main C flux from terrestrial ecosystems to the atmosphere [1], [2] and is therefore one of the major components to consider in understanding ecosystem-atmosphere C exchange [3]. Carbon flux from soils has received growing attention in recent years, due to elevating atmospheric CO_2_ concentration causing increasing air temperature [4]–[6]. Most SR measurements are conducted during the plant-growing season [6], [7] when instantaneous flux rates are much higher than during colder seasons. However, Monson et al. [8] suggested that winter soil respiration could offset a major portion of the carbon fixed during the growing season, and thus, significantly contribute to the annual carbon cycling. Significant winter CO_2_ flux, with a long snow-cover period, has been reported in the more productive meadow and forest ecosystems [9]–[11]. Mid-latitude ecosystems, on the other hand, are dominated by a shorter winter season and a thinner snow depth. These mid-latitude ecosystems are considered to be major terrestrial carbon sinks in the northern hemisphere [12]. However, little is known about the winter SR and its contribution to annual soil C flux in different mid-latitude ecosystems, which may yield inaccurate regional and global C budget predictions [13], [14].

SR is a composite process shown, through field investigations, to be influenced by a multitude of environmental factors [15]–[16]. To date, most studies have based on SR rate predictions on the relationship between soil CO_2_ flux and soil temperature (ST) and moisture (SM) [17]–[19]. Regional scale microclimates induced by topography and vegetation cover can affect SR rate by constraining microsite factors, such as ST and SM [19]. Therefore, there is clearly much to be learned about the major factors that control SR at the regional scale level across different ecosystems [20].

The Taihang Mountainous region covers approximately 42% of north China [21]. The vegetation of this area is a mosaic of grass, shrubs and plantation. The grass vegetation is dominated by the mixed drought-resistant species of *Artemisia sacrorum*, *Bothriochloa ischaemum* and *Themeda japonica*; shrub vegetation is dominated by *Vitex negundo var. heterophylla*; and plantation vegetation is dominated by *Populus tomatosa* species [21], [22]. The region is characterized by short, cold, and dry winters, lasting approximately three months long from December to February, with varied snow cover. We hypothesized that, with all other factors being equal, shorter winter SR result in reduced contribution to annual soil C flux. This hypothesis was tested by through field measurements of SR in three mountainous sites: grassland, shrubland, and plantation. The magnitude of winter SR rate and its contribution to annual soil C flux was compared among the different sites. In addition, the present study also investigated the relationships among SR and ST and SM.

## Materials and Methods

### Ethics Statement

All necessary permits were obtained for the described field studies. We carried out the study based on the Hilly Ecosystem Experimental Station of Taihang Mountain, which belongs to the Center for Agricultural Resources Research, Institute of Genetics and Developmental Biology, Chinese Academy of Science. We obtained permissions to use the sample plots from the station and institute. Our study inflicted no harm to the environment and did not involve endangered or protected species.

### Site Description

**Figure 1:**
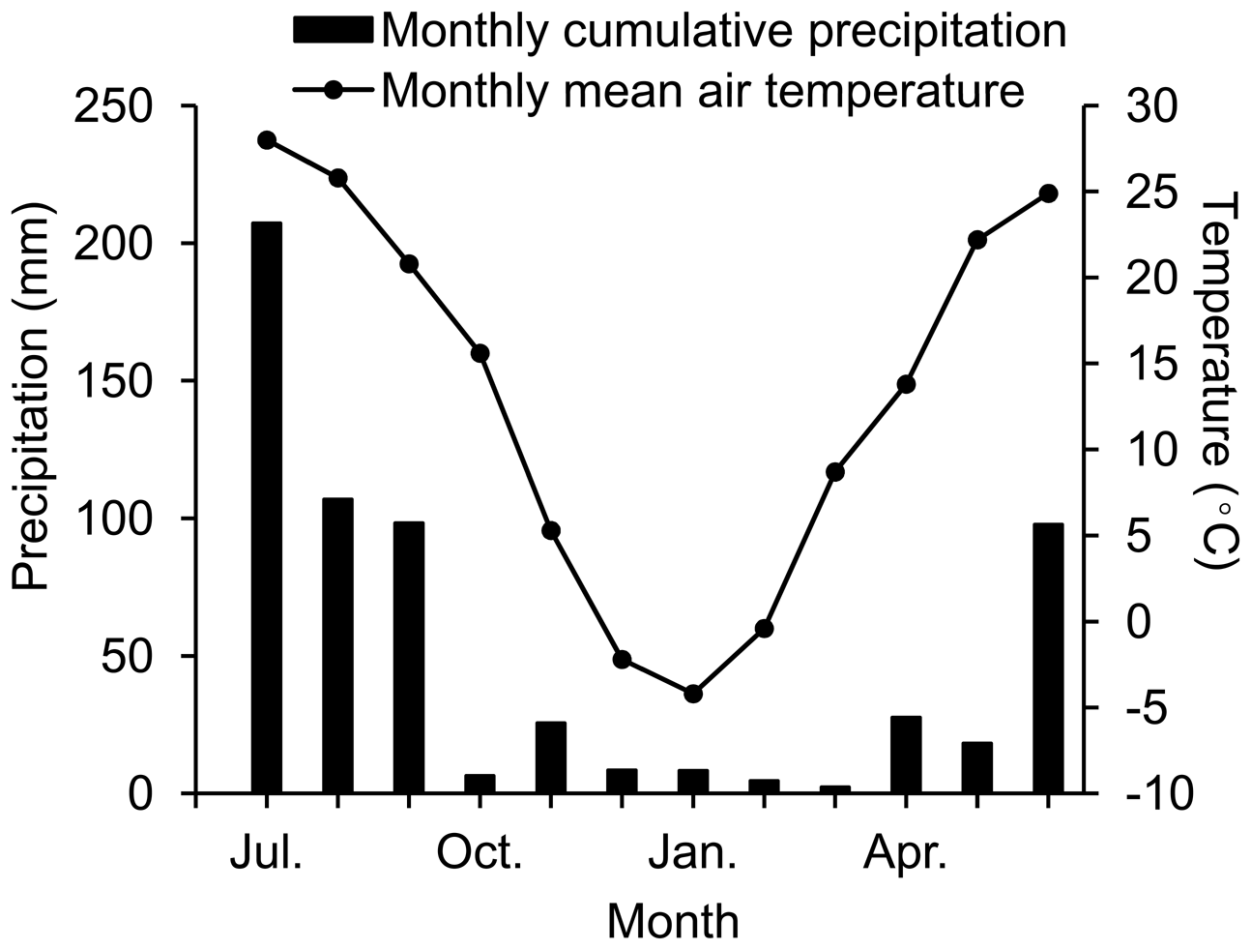
Average monthly climatic data of the study region during experimental periods from July 2012 to June 2013.

The field-study was conducted in the Taihang Mountain Ecological Experimental Station of the Chinese Academy of Science, Hebei Province. The long-term annual mean air temperature of this region is 13°C, with January (–4°C mean temperature) and July (26°C mean temperature) as the coldest and warmest months, respectively [22], [23]. Mean annual precipitation is 560 mm, of which 70% falls between June and September. Monthly cumulative precipitation and mean air temperature during the study period are shown in Figure 1. The top 40-cm soil layer is classified as Luvisol (FAO-UNESCO 1974). In July 2012, three 10 m×10 m plots were randomly established within each grassland, shrubland and plantation site. The three different sites were separated by a minimum of 1000 m. Stand and soil characteristics of the three sites are summarized in Table 1. No sites from which SR was measured were irrigated.

**Table 1:** Summary of characteristics of the different sites.

Characteristics	Grassland	Shrubland	Plantation
Latitude	N37°54′17.08″	N37°54′12.00″	N37°54′14.12″
Longitude	E114°14′55.37″	E114°14′50.82″	E114°15′18.16″
Altitude (m)	374	387	381
Plot size (m^2^)	100	100	100
ST (°C)	−2.6–29.1	−3.8–30.0	−5.3–27.0
SM (%)	0.6–30.2	0.4–15.3	0.3–24.0
SOC (g kg^−1^)	15.0±1.9	23.1±1.4	22.4±2.5
SBD (g cm^−3^)	1.47±0.07	1.38±0.08	1.32±0.05
Vegetation type	*A. sacrorum* *B. ischcemum* *T. japonica*	*V. negundo var. heterophylla*	*P. tomatosa*

ST and SM are range of mean soil temperature and soil moisture, respectively at 10 cm depth, during the experimental period; SOC represents soil organic carbon in the top 20 cm depth; SBD means soil bulk density.

### Experimental Design and Measurement

In each plot, three 100 cm^3^ soil columns were randomly taken from 0 to 20 cm depth to determine the soil bulk density. Meanwhile, a soil subsample of approximately 200 g was collected at the same locations and brought back to the lab for organic C content analysis. Soil total organic C content was determined using the potassium dichromate method. All methods described above are conducted based on Forestry Standards ‘Observation Methodology for Long-term Forest Ecosystem Research’ of the People’s Republic of China.

Within each plot, three PVC collars (20 cm inner diameter and 10 cm height) were inserted into the soil with 3 cm exposed above the soil surface, and remained permanently installed throughout the experiment. Green plants and litter inside the collar were removed carefully before SR measurements. SR was measured using an automated soil CO_2_ flux systems (Li-8100, Li-Cor Inc., Lincoln, Nebraska, USA) equipped with a 20 cm survey chamber (Model 8100-103). This system works as a dynamic closed chamber, which was manually controlled using a LifeDrive PDA (Palm Inc., Sunnyvale, CA, USA).

SR was measured on clear days once every month from July 2012 to June 2013. At the time of SR measurements, ST and SM at 10 cm depth were measured automatically using the soil temperature probe and moisture sensor equipped with the LI-8100 system. In order to minimize daily variation in SR measurements due to diurnal changes in SR, measurements were made between 8∶00 and 11∶00 h. Additionally, to assess seasonal effects on diurnal patterns, SR rates were measured every 2 h from 8∶00 to 18∶00 in July and October in 2012, and in January and April in 2013, representing spring, summer, autumn, and winter, respectively. The winter length in the present study was defined as the period during which mean diel ST at 5 cm was continuously <0.5°C [11], and occurred for 3 months from December to February. Respiration rates for each measurement for each site were calculated as means of the nine collars within each site.

### Dependence of SR on ST and SM

Exponential and polynomial functions, based on the measurements collected, were established to describe the relationships between SR and ST and between SR and SM:
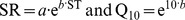
(1)




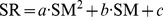
(2)where *a*, *b* and *c* are fitted constants; Q_10_ is the temperature sensitivity of SR; ST and SM are the soil temperature (°C) and soil moisture (%) at 10 cm depth, respectively.

Non-linear regression analysis was used to express SR against ST and SM:
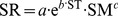
(3)where *a*, *b* and *c* are fitted constants.

### Scaling for Annual and Winter Soil C Flux

A further estimate of winter and annual soil CO_2_ flux for each site was obtained by interpolating measured SR between respective sampling dates for each seasonal measurement period of the year, and then computing the sum to obtain the “measured” winter or annual values [20], [24] as follows:
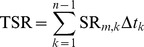
(4)where Δ*t_k_* = *t_k_*_+1_– *t_k_*, which is the numbecr of days between each field measurement within the season; TSR is total SR during the prediction period (annual/winter); SR*_m_*_,*k*_ is the average SR rate over the interval *t_k_*_+1_– *t_k_* recorded by the LI-8100 soil CO_2_ flux system; and *n* is the number of soil CO_2_ flux measurements made within each season.

In order to confirm whether measuring winter SR is important, the equation describing the relationship between SR and ST (Equation (1)) during the growing season was also used to estimate the “modeled” annual SR rates based on actual ST measurements. Equation (4) was also used to calculate the “modeled” annual values.

### Statistical Analysis

A one-way ANOVA was used to compare differences in mean values of SR, ST and SM among the three sites. Pairwise *t* tests were carried out to compare the “measured” and “modeled” annual values. All statistical linear and nonlinear regression analyses, multiple comparisons including the one-way ANOVA were performed with a significance level of 0.05 using the SPSS 15.0 software (SPSS Inc., Chicago, IL, USA).

## Results

### Temporal Dynamics of SR

**Figure 2:**
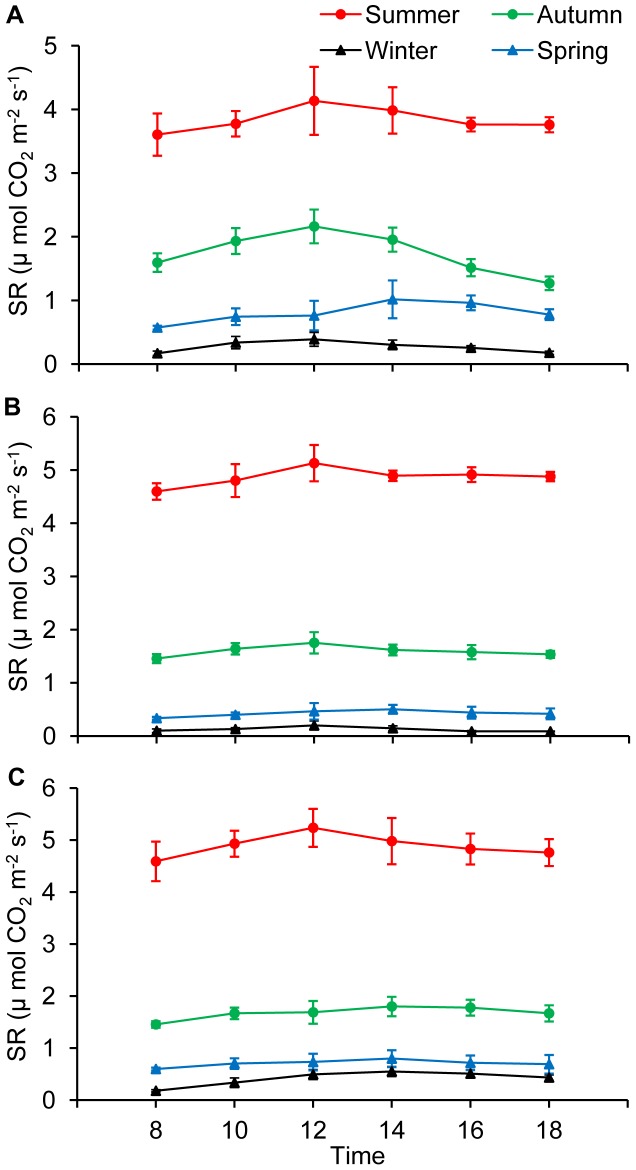
Daily and seasonal variations in soil respiration (SR) in the grassland (A), shrubland (B) and plantation (C) sites, respectively. Note: Time of *x*-axis is Chinese Standard Time (UTC+8). Error bars are standard error of means (n = 9).

The diurnal pattern of SR for all three sites was described by asymmetric single-peak curves (Figure 2). On each day, the soil CO_2_ flux increased gradually from 8∶00, achieving a maximum rate at 12∶00 or 14∶00, and then decreasing. The morning SR followed the increasing trend of ST at 10 cm depth, but decreased more quickly than the temperature in the afternoon. In autumn, the diurnal range of SR in the grassland was larger than in shrubland and plantation. There were no obvious daily fluctuations among the three sites in winter or spring.

**Figure 3:**
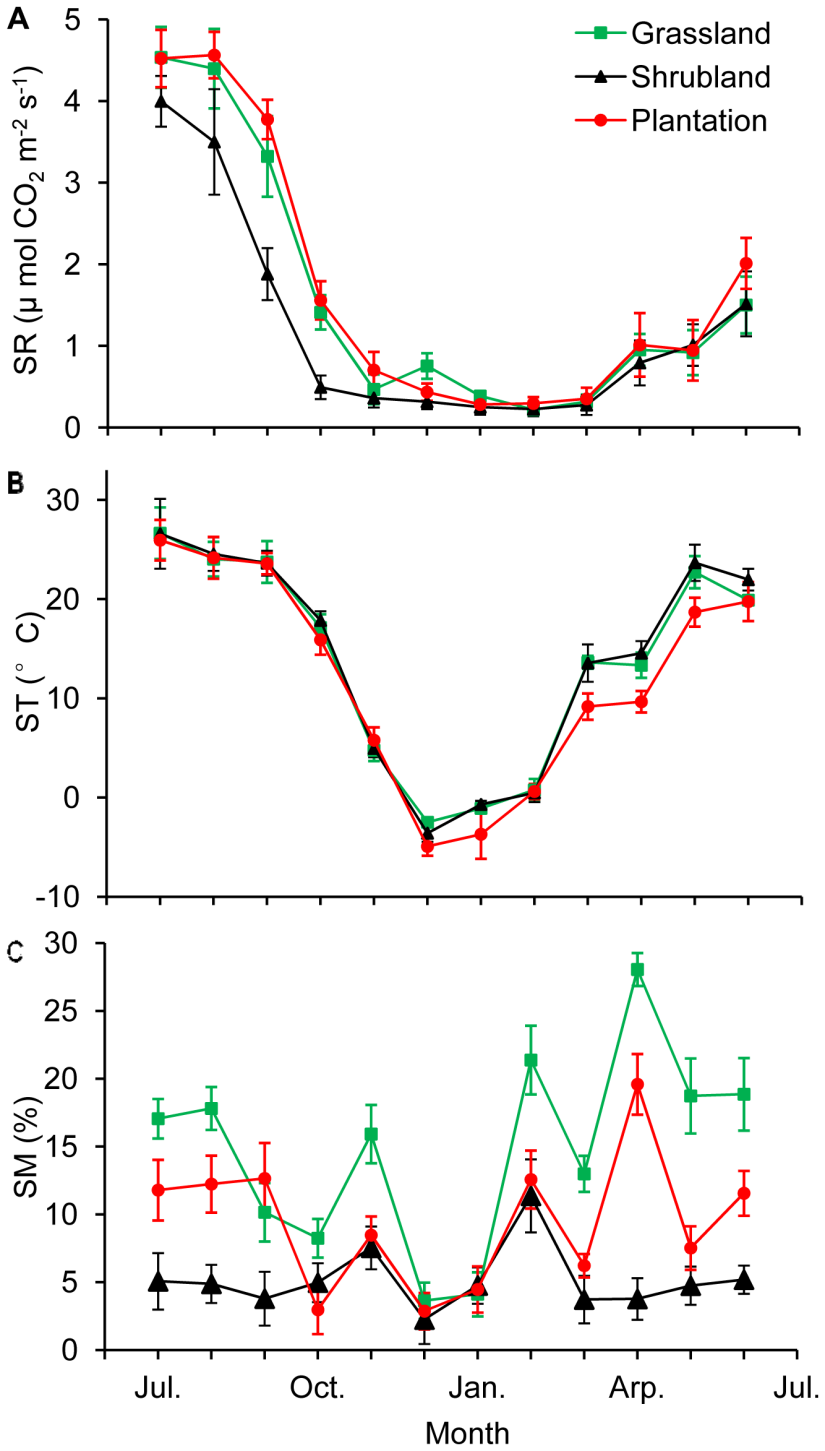
Temporal variations in SR (A), ST at 10 cm depth (B) and SM at 10 cm depth (C) for the three different sites. SR, ST and SM represents soil respiration, soil temperature and soil moisture, respectively. Error bars are standard error of means (n = 9).

The within year temporal dynamics of soil CO_2_ flux were largely the same for the three sites (Figure 3A). From October, SR declined steadily until the next March, and then began to increase in April (the beginning of the growing season). The lowest monthly SR rate was 0.22 µmol m^−2^ s^−1^, and occurred in the grassland in February. SR rate peaked in July or August in all of the sites, with the maximum rate of 4.56 µmol m^−2^ s^−1^ in the plantation in August.

### Dependence of SR on ST and SM

ST varied distinctly with season paralling the seasonality observed in SR (Figure 3B). Over the whole year, a significant (*p*<0.001) exponential relationship was found between SR and ST for different sites. ST alone explained 73.8 to 91.8% of the within year temporal variability in SR rates (Table 2). The Q_10_ values of SR against ST at 10 cm ranged from 3.60 to 4.90 among different sites. The polynomial function provided the best fit for the relationship between SR and SM. However, the SM-based models could only explain 13.0 to 26.4% of the variation in SR (Table 2). The combined use of ST and SM functions explained 70.6 to 81.9% of the variation in SR, indicating that the inclusion of SM did not improve the explanation of SR compared with the regressions based on ST only.

**Table 2:** Effects of soil temperature and soil moisture on the variation in soil respiration rate of different sites during the experimental period.

Ecosystem	Regression equation											
	SR = *a*·e*^b·^* ^ST^				SR = *a*·SM^2^+ *b*·SM+*c*				SR = *a*·e*^b·^* ^ST^·SW^c^			
	*a*	*b*	*R* ^2^	Q_10_	*a*	*b*	*c*	*R* ^2^	*a*	*b*	*c*	*R* ^2^
Grassland	0.120	0.135	0.738***	3.86	−0.010	0.298	−0.244	0.134*	1.110	0.093	−0.541	0.706***
Shrubland	0.052	0.167	0.775***	4.90	−0.038	0.501	−0.017	0.130*	0.154	0.084	0.282	0.758***
Plantation	0.176	0.128	0.913***	3.60	−0.021	0.557	−1.213	0.264**	0.361	0.088	0.0048	0.819***

SR is monthly mean soil respiration (µmol m^−2^ s^−1^); ST and SM denote soil temperature (°C) and soil moisture (%) at 10 cm depth, respectively; *a*, *b* and *c* are fitted constants; Q_10_ is the temperature sensitivity of SR.

*means *p*<0.1;

**means *p*<0.05;

***means *p*<0.001.

### Winter SR Rates and their Contributions to Annual soil C Flux

**Figure 4:**
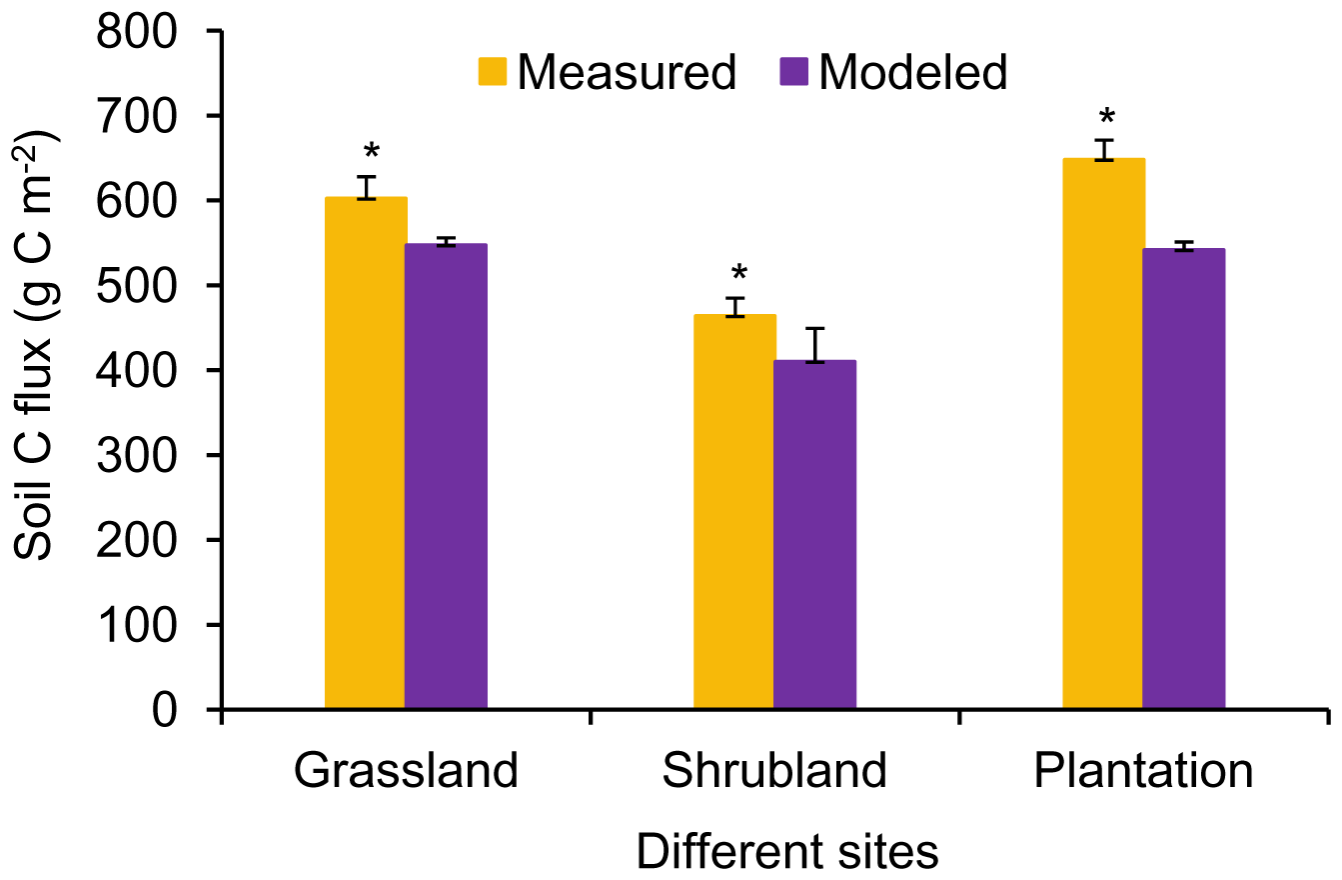
Comparison of “measured” and “modeled” annul soil C flux from the different sites. *denotes statistical significance using a pairwise *t* test comparing “measured” with “modeled” annual values at each site.

**Figure 5:**
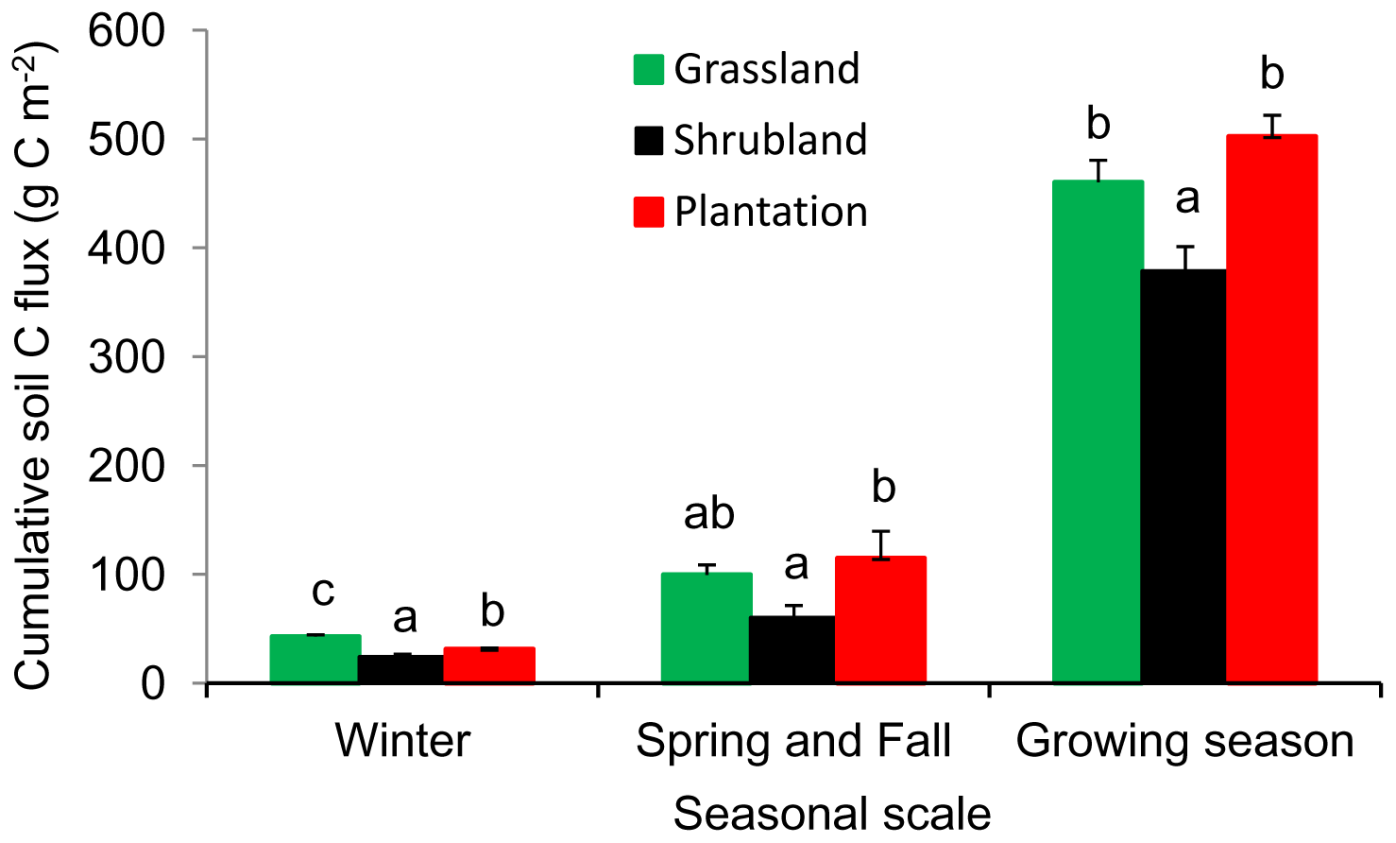
Cumulative soil C flux (g C m^−2^) for Winter (December-February), Spring and Fall (March-April and October-November), and Growing season (May-September) in the three different sites. Error bars are standard error of means (n = 3). Different letters denote significant differences as determined by Tukey’s HSD test.

Mean monthly soil CO_2_ flux rates were 1.58, 1.22 and 1.70 µmol m^−2^ s^−1^, and the “measured” annual soil C fluxes were 602.7, 464.1 and 648.5 g C m^−2 ^for the grassland, shrubland and plantation sites, respectively (Figure 4). Mean winter soil CO_2_ flux rate ranged from 0.26 to 0.45 µmol m^−2^ s^−1^ depending on site, which made up 10.6 to 15.6% of the mean growing season soil CO_2_ flux. Winter soil C flux, from December 2012 to February 2013, ranged from 24.6 to 42.8 g C m^−2^ (Figure 5). In the Spring and Fall seasons, mean soil CO_2_ flux rate ranged from 0.48 to 0.91 µmol m^−2^ s^−1^ depending on site, and made up 20.2 to 28.7% of the mean growing season soil CO_2_ flux. Total non-growing season (Winter and Spring and Fall) soil C flux ranged from 85.1 to 146.1 g C m^−2^ (Figure 5). The contributions of winter (December to February) and total non-growing season (November to April) soil C flux to annual soil C flux were 4.8 to 7.1% and 18.3 to 23.6%, respectively, depending on site. In addition, the pairwise *t* test showed that the “measured” annual value was significantly higher than the “modeled” annual value for each site (*p*<0.01) (Figure 4).

## Discussion

### Winter SR

Due to the assumption that winter SR was very small compared to growing season respiration, previous to this study, most SR measurements had been conducted during the growing season [3], [7], [25], and the annual soil CO_2_ flux has generally been calculated by extrapolation of growing-season empirical functions [26], [27]. However, winter SR can lead to significant carbon losses. For instance, winter soil CO_2_ flux accounted for 3.5 to 17% of annual SR estimates in mid-latitude sites [20], [24], [28], [29]. Therefore, winter SR should not be ignored when attempting to quantify and understand the annual carbon balance of terrestrial ecosystems [20], [24], [25].

Our measured mean winter SR rates (0.26–0.45 µmol m^−2^ s^−1^) were consistent with the results from a study conducted in a forest-steppe ecotone in north China (0.15–0.26 µmol m^−2^ s^−1^) [20] and another study on three vegetation types in the Yellow River Delta of China (0.17–0.60 µmol m^−2^ s^−1^) [30]. Our results were generally lower than other studies conducted in coniferous forests, including those by McDowell et al. [28] (2000) from the mid-elevation forests in northern Idaho (0.8 µmol m^−2^ s^−1^), and Schindlbacher et al. [29] in a mountain forest in Austria (0.64 µmol m^−2^ s^−1^). Higher surface soil organic matter and biological activity in forests may contribute to the higher winter SR rates than those of the present study. In addition, the thickness and duration of snow cover influence the subsurface ST, which may further affect winter SR rates [30]. The snow cover was generally between 0 and 10 cm and the duration of snow cover was very short in the present study region, both of which could also contribute to low winter ST and correspondingly low soil CO_2_ flux in this study region.

Changes in winter SR in response to climate change have the potential to substantially reduce the net carbon sink in terrestrial ecosystems [9], [31]. The contribution of winter SR to annual soil C flux varies with sites and may be affected by many factors, e.g. relative length of winter and growing season, temperature, snow cover, vegetation and soil properties [8], [11], [25]. Brooks et al. [9] reviewed published studies showing that, on average, 50% of the growing season carbon uptake is respired during the winter. In this study, the contribution of winter SR to annual soil C flux (4.8–7.1%) among different sites was consistent with reported results in a forest-steppe ecotone in northeast China (3.5–7.3%) [20] and in crop rotation fields of northeast China (5.1–7.2%) [24]. Moreover, we also computed an SR-ST relationship for the growing season, and then used this equation to calculate the “modeled” annual soil C flux based on the actual measured ST. The “measured” annual value was significantly higher than the “modeled” annual value, which seems to contradict the hypothesis that shorter winter result in reduced winter contribution to annual C flux. Though our results highlighted the importance of winter SR to annual soil C flux in mid-latitude sites of mountainous region, where winter are short, they can’t be generalized to broad scales without further sampling.

### Dependence of SR on ST and SM

In this study, the exponential relationship between SR and ST for the three different sites was consistent with other field SR studies [33]–[35]. The ST-based model explained temporal variation in SR of all three sites very well during the experimental period (Table 2). Previous studies suggested that SR rates increased with an increase in ST [36], [37], and this effect was clearly evident in this study as well. In addition to ST, SM has also been recognized as an important factor controlling the variations in SR [17], [38], [39]. In this study, polynomial equations best fitted the SR with SM relationship. However, the fits of the SM-based models were very low (13.0–26.4%) (Table 2). The combined use of ST and SM functions did not improve model fitting compared with the functions based on ST alone, thus suggesting that ST was a good surrogate for estimating annual SR in the three studied sites of Middle Taihang Mountain. However, previous studies have indentified other factors, such as litter and fine roots, which impact SR [6], [40], [41].

The Q_10_ is commonly used to express the relationship between SR and ST. The annual Q_10_ values ranged from 3.60 to 4.60 in this study, which is consistent with the range (1.12–5.53) reported for other temperate ecosystems [6], [19], [20]. The differences in Q_10_ values among sites point to site effects on the response of SR to ST. In addition, the seasonal variations in Q_10_ may reflect confounding effects of seasonal changes in physiological activities induced by root phenology, microbial biomass and other factors [1], [42]. Understanding the sensitivity of SR to temperature change and other soil factors makes it possible to improve accuracy of evaluation of the response of terrestrial carbon balance to climatic change [43].

### Summary

We measured SR rates during the growing season and non-growing season throughout the year in north China. This study showed that the winter (December to February) and non-growing season (October to April) SR accounted for 4.8 to 7.1% and 18.3 to 23.6%, respectively, of annual soil C flux in the study area. ST was a good proxy for estimating within year temporal variation in SR. This study found that ignoring the winter SR would lead to underestimates of C loss potential in temperate sites. Our results presented here are consistent with other studies indicating that winter soil C flux plays an important role in the global carbon budget.

## References

[1] Luan J, Liu S, Zhu X, Wang J, Liu K (2012) Roles of biotic and abiotic variables in determining spatial variation of soil respiration in secondary oak and planted pine forests. Soil Biology and Biochemistry 44: 143–150.

[2] Song X, Yuan H, Kimberley MO, Jiang H, Zhou G, et al (2013) Soil CO_2_ flux dynamics in the two main plantation forest types in subtropical China. Science of Total Environment 444: 363–368. 10.1016/j.scitotenv.2012.12.006 23280294

[3] Arevalo CBM, Bhatti JS, Chang SX, Jassal RS, Sidders D (2010) Soil respiration in four different land use systems in north central Alberta, Canada. Journal of Geophysical Research 115: G01003.

[4] Dorrepaal E, Toet S, van Logtestijn RSP, Swart E, van de Weg MJ, et al (2009) Carbon respiration from subsurface peat accelerated by climate warming in the subarctic. Nature 460: 616–619.

[5] Bond-Lamberty B, Thomson A (2010) Temperature-associated increases in the global soil respiration record. Nature 464: 579–582. 20336143 10.1038/nature08930

[6] Davidson EA, Richardson AD, Savage KE, Hollinger DY (2006) A distinct seasonal pattern of the ratio of soil respiration to total ecosystem respiration in a spruce-dominated forest. Global Change Biology 12: 230–239.

[7] Chen Q, Wang Q, Han X, Wan S, Li L (2010) Temporal and spatial variability and controls of soil respiration in a temperate steppe in northern China. Global Biogeochemical Cycles 24: GB2010.

[8] Monson RK, Sparks JP, Rosenstiel TN, Scott-Denton LE, Huxman TE, et al (2005) Climatic influences on net ecosystem CO2 exchange during the transition from wintertime carbon source to springtime carbon sink in a high-elevation, subalpine forest. Oecologia 146: 130–147. 16091970 10.1007/s00442-005-0169-2

[9] Brooks PD, McKnight D, Elder K (2004) Carbon limitation of soil respiration under winter snowpacks: potential feedbacks between growing season and winter carbon fluxes. Global Change Biology 11: 231–238.

[10] Du E, Zhou Z, Li P, Jiang L, Hu X, et al (2013) Winter soil respiration during soil-freezing process in a boreal forest in Northeast China. Journal of Plant Ecology 6: 349–357.

[11] Grogan P, Jonasson S (2006) Ecosystem CO_2_ production during winter in a Swedish subarctic region: the relative importance of climate and vegetation type. Global Change Biology 12: 1479–1495.

[12] Schimel D, Kittel TGF, Running S, Monson R, Turnipseed A, et al (2002) Carbon sequestration studied in western U.S. mountains. Eos, Transactions American Geophysical Union 83: 445–449.

[13] Mariko S, Nishimura N, Mo W, Matsui Y, Kibe T, et al (2000) Winter CO2 flux from soil and snow surfaces in a cool-temperate deciduous forest, Japan. Ecological Research 15: 363–372.

[14] Uchida M, Mo W, Nakatsubo T, Tsuchiya Y, Horikoshi T, et al (2005) Microbial activity and litter decomposition under snow cover in a cool-temperate broad-leaved deciduous forest. Agricultural and Forest Meteorology 134: 102–109.

[15] Gaumont-Guay D, Black TA, Griffis TJ, Barr AG, Jassal RS, et al (2006) Interpreting the dependence of soil respiration on soil temperature and water content in a boreal aspen stand. Agricultural and Forest Meteorology 140: 220–235.

[16] Li LJ, You MY, Shi HA, Ding XL, Qiao YF, et al (2013) Soil CO_2_ emissions from a cultivated Mollisol: Effects of organic amendments, soil temperature, and moisture. European Journal of Soil Biology 55: 83–90.

[17] Cook FJ, Orchard VA (2008) Relationships between soil respiration and soil moisture. Soil Biology and Biochemistry 40: 1013–1018.

[18] Pavelka M, Acosta M, Marek MV, Kutsch W, Janous D (2007) Dependence of the Q_10_ values on the depth of the soil temperature measuring point. Plant and Soil 292: 171–179.

[19] Li HJ, Yan JX, Yue XF, Wang MB (2008) Significance of soil temperature and moisture for soil respiration in a Chinese mountain area. Agricultural and Forest Meteorology 148: 490–503.

[20] Wang W, Peng S, Wang T, Fang J (2010) Winter soil CO_2_ efflux and its contribution to annual soil respiration in different ecosystems of a forest-steppe ecotone, north China. Soil Biology and Biochemistry 42: 451–458.

[21] Han S, Yang Y, Fan T, Xiao D, Moiwo JP (2012) Precipitation-runoff processes in Shimen hillslope micro-catchment of Taihang Mountain, north China. Hydrological Processes 26: 1332–1341.

[22] Liu XP, Zhang WJ, Hu CS, Tang XG (2013) Soil greenhouse gas fluxes from different tree species on Taihang Mountain, North China. Biogeosciences Discussions 10: 11037–11076.

[23] Cao J, Liu C, Zhang W, Han S (2013) Using temperature effect on seepage variations as proxy for phenological processes of basin-scale vegetation communities. Hydrological Processes 27: 360–366.

[24] Shi X, Zhang X, Yang X, Drury CF, McLaughlin NB, et al (2012) Contribution of winter soil respiration to annual soil CO_2_ emission in a Mollisol under different tillage practices in northeast China. Global Biogeochemical Cycles 26: GB2007.

[25] Wang T, Ciais P, Piao SL, Ottlé C, Brender P, et al (2011) Controls on winter ecosystem respiration in temperate and boreal ecosystems. Biogeosciences 8: 2009–2025.

[26] Lellei-Kovács E, Kovács-Láng E, Botta-Dukát Z, Kalapos T, Emmett B, et al (2011) Thresholds and interactive effects of soil moisture on the temperature response of soil respiration. European Journal of Soil Biology 47: 245–255.

[27] Tang J, Bolstad PV, Desai AR, Martin JG, Cook BD, et al (2008) Ecosystem respiration and its components in an old-growth forest in the Great Lakes region of the United States. Agricultural and Forest Meteorology 148: 171–185.

[28] McDowell NG, Marshall JD, Hooker TD, Musselman R (2000) Estimating CO_2_ flux from snowpacks at three sites in the Rocky Mountains. Tree Physiology 20: 745–753. 12651510 10.1093/treephys/20.11.745

[29] Schindlbacher A, Zechmeister-Boltenstern S, Glatzel G, Jandl R (2007) Winter soil respiration from an Austrian mountain forest. Agricultural and Forest Meteorology 146: 205–215.

[30] Han G, Yu J, Li H, Yang L, Wang G, et al (2012) Winter soil respiration from different vegetation patches in the Yellow River Delta, China. Environmental Management 50: 39–49. 22576142 10.1007/s00267-012-9869-7

[31] Elberling B (2007) Annual soil CO_2_ effluxes in the High Arctic: The role of snow thickness and vegetation type. Soil Biology and Biochemistry 39: 646–654.

[32] Michaelson GJ (2003) Soil organic carbon and CO_2_ respiration at subzero temperature in soils of Arctic Alaska. Journal of Geophysical Research 108.

[33] Mo W, Lee M-S, Uchida M, Inatomi M, Saigusa N, et al (2005) Seasonal and annual variations in soil respiration in a cool-temperate deciduous broad-leaved forest in Japan. Agricultural and Forest Meteorology 134: 81–94.

[34] Vargas R, Allen MF (2008) Environmental controls and the influence of vegetation type, fine roots and rhizomorphs on diel and seasonal variation in soil respiration. New Phytologist 179: 460–471. 19086292 10.1111/j.1469-8137.2008.02481.x

[35] You W, Wei W, Zhang H, Yan T, Xing Z (2013) Temporal patterns of soil CO_2_ efflux in a temperate Korean Larch (*Larix olgensis* Herry.) plantation, Northeast China. Trees 27: 1417–1428.

[36] Kellman L, Beltrami H, Risk D (2006) Changes in seasonal soil respiration with pasture conversion to forest in Atlantic Canada. Biogeochemistry 82: 101–109.

[37] Wu X, Brüggemann N, Gasche R, Shen Z, Wolf B, et al (2010) Environmental controls over soil-atmosphere exchange of N_2_O, NO, and CO_2_ in a temperate Norway spruce forest. Global Biogeochemical Cycles 24: GB2012.

[38] Jiang H, Deng Q, Zhou G, Hui D, Zhang D, et al (2013) Responses of soil respiration and its temperature/moisture sensitivity to precipitation in three subtropical forests in southern China. Biogeosciences 10: 3963–3982.

[39] Zhang LH, Chen YN, Zhao RF, Li WH (2010) Significance of temperature and soil water content on soil respiration in three desert ecosystems in Northwest China. Journal of Arid Environments 74: 1200–1211.

[40] Zhou Z, Zhang Z, Zha T, Luo Z, Zheng J, et al (2013) Predicting soil respiration using carbon stock in roots, litter and soil organic matter in forests of Loess Plateau in China. Soil Biology and Biochemistry 57: 135–143.

[41] Zimmermann M, Meir P, Bird M, Malhi Y, Ccahuana A (2009) Litter contribution to diurnal and annual soil respiration in a tropical montane cloud forest. Soil Biology and Biochemistry 41: 1338–1340.

[42] Luo J, Chen YC, Wu YH, Shi PL, She J, et al (2012) Temporal-spatial variation and controls of soil respiration in different primary succession stages on Glacier Forehead in Gongga Mountain, China. PLoS ONE 7: e42354. 22879950 10.1371/journal.pone.0042354 PMC3412854

[43] Peng S, Piao S, Wang T, Sun J, Shen Z (2009) Temperature sensitivity of soil respiration in different ecosystems in China. Soil Biology and Biochemistry 41: 1008–1014.

